# *Pythium insidiosum:* an emerging pathogen that is easily misdiagnosed and given treatment as a fungus

**DOI:** 10.3389/fcimb.2024.1430032

**Published:** 2024-08-29

**Authors:** Liuyang Hu, Xiulu Huang, Ngan Hung Yee, Huixia Meng, Li Jiang, Liang Liang, Xingchun Chen

**Affiliations:** ^1^ Department of Laboratory Medicine, The People’s Hospital of Guangxi Zhuang Autonomous Region, Guangxi Academy of Medical Sciences, Nanning, China; ^2^ Department of endocrinology, The People’s Hospital of Guangxi Zhuang Autonomous Region, Guangxi Academy of Medical Sciences, Nanning, China; ^3^ Department of Microbiology, Queen Mary Hospital, Hong Kong, Hong Kong SAR, China; ^4^ Department of Microbiology, Guangxi Jinyu Medical Laboratory Co., Ltd., Nanning, China; ^5^ Department of Ophthalmology, The People’s Hospital of Guangxi Zhuang Autonomous Region, Guangxi Academy of Medical Sciences, Nanning, China

**Keywords:** pythiosis, *Pythium insidiosum*, pythium keratitis, cutaneous and subcutaneous pythiosis, antimicrobial agent susceptibility

## Abstract

**Background:**

*Pythium insidiosum* (*P. insidiosum*) is the causative agent of pythiosis, an infectious disease with a high morbidity and fatality rate. Pythiosis cases have increased dramatically during the past ten years, particularly in tropical and subtropical areas. Sadly, microbiologists and medical professionals know very little about pythiosis, and the disease is frequently challenging to identify. It is frequently misdiagnosed as a fungal infection.

**Methods:**

We report two cases of pythiosis, one was *Pythium* keratitis, the other was cutaneous pythiosis. The patient with corneal infection had no underlying disease, while the patient with cutaneous pythiosis had a history of liver cirrhosis, diabetes, and psoriasis. The corneal sample and subcutaneous pus were sent for metagenomic Next-Generation Sequencing (mNGS). To further diagnose the isolated strain, *P. insidiosum* zoospores were induced to produce by co-incubation with sterile grass leaves in sterile pond water. Their zoospores were used as an inoculum for drug susceptibility testing by disk diffusion and broth microdilution method.

**Results:**

The mNGS of two cases were reported as *P. insidiosum.* Zoospores were produced after incubation 48h. The zoospores were collected for drug susceptibility assay. All antifungal drugs, antibacterial drugs of β-Lactams, vancomycin, levofloxacin, ciprofloxacin, gentamicin, trimethoprim-sulfamethoxazole, clindamycin have no inhibitory activity against *P. insidiosum in vitro*. Minocycline, tigecycline, linezolid, erythromycin and azithromycin have significant *in vitro* activity against *P. insidiosum*. Based on the susceptibility results, the drug was changed from itraconazole to linezolid and minocycline, along with multiple debridements and drainage for cutaneous pythiosis. The patient was discharged after 24 days of treatment.

**Conclusions:**

Early and accurate identification, combined with aggressive surgical debridement and appropriate drug therapy, can greatly improve patient managements. Conventional culture and zoospore induction remain gold standard for diagnosis; however, DNA-based method should be performed simultaneously. The drug susceptibility testing provides profound effects on proper drug selection against *P. insidiosum*.

## Introduction

Pythiosis caused primarily by the fungus-like aquatic oomycete *P. insidiosum*, is an emerging but uncommon infection in China. It is the most common species in the Genus *Pythium* that can cause disease in humans and animals. Pythiosis mainly occurs in tropical and subtropical regions, especially in horses, dogs, and humans (Kaufman, 1998). Infection occurs when the host comes into contact with water contaminated with zoospores after skin damage. The zoospores attach and germinate as hyphae into injured tissue. Agricultural-related activities or water-related recreational activities are considered triggering factors for human pythiosis. *P. insidiosum* grows by forming filamentous structures similar to fungal hyphae, with broad, irregularly branched, ribbon-like hyphae that can fold easily. Morphologically, it resembles hyphae of *Mucorales*, although *P. insidiosum* is not a fungus but a protist. It shows similarities to fungal infections clinically and microbiologically, leading to the widespread use of antifungal drugs for treatment, resulting in high treatment failure rates. Despite some successful reports of antifungal drug treatment (Shenep et al., 1998; Heath et al., 2002), many studies have shown their ineffectiveness. *P. insidiosum* does not synthesize ergosterol (Sathapatayavongs et al., 1989; Krajaejun et al., 2006b), which is the target of most antifungal drugs. Recent *in vitro* and *in vivo* studies suggested that certain antibiotics may be more suitable for treating *P. insidiosum* infections compared to antifungal drugs. Pythiosis can cause four clinical manifestations: cutaneous and subcutaneous infections, vascular pythiosis, corneal ulcer, and systemic infections (Gaastra et al., 2010). Pythiosis is most commonly reported in South and Southeast Asia, although cases of pythiosis have been reported worldwide (Chitasombat et al., 2020). Recently, an outbreak of *pythium* keratitis during the rainy season (Thanathanee et al., 2013), as well as severe skin and subcutaneous pythiosis have been reported in China and North America (Perkins et al., 2022; Zhang et al., 2022). Initially, they were misdiagnosed as fungal infections. Conventional culture and zoospores induction remain the gold standard for diagnosis; however, DNA-based methods should also be performed concurrently. Curative surgery, amputation, and surgical debridement of the lesions are the most commonly used and effective treatment methods, but recurrent rates are high (45%) (Gaastra et al., 2010).

We report two cases of pythiosis which were initially misdiagnosed as fungal infections. We propose new diagnostic, drug susceptibility test, and treatment methods to minimize misdiagnosis and ineffective treatment.

## Materials and methods

### Case presentation

#### Patient 1

**Figure 1:**
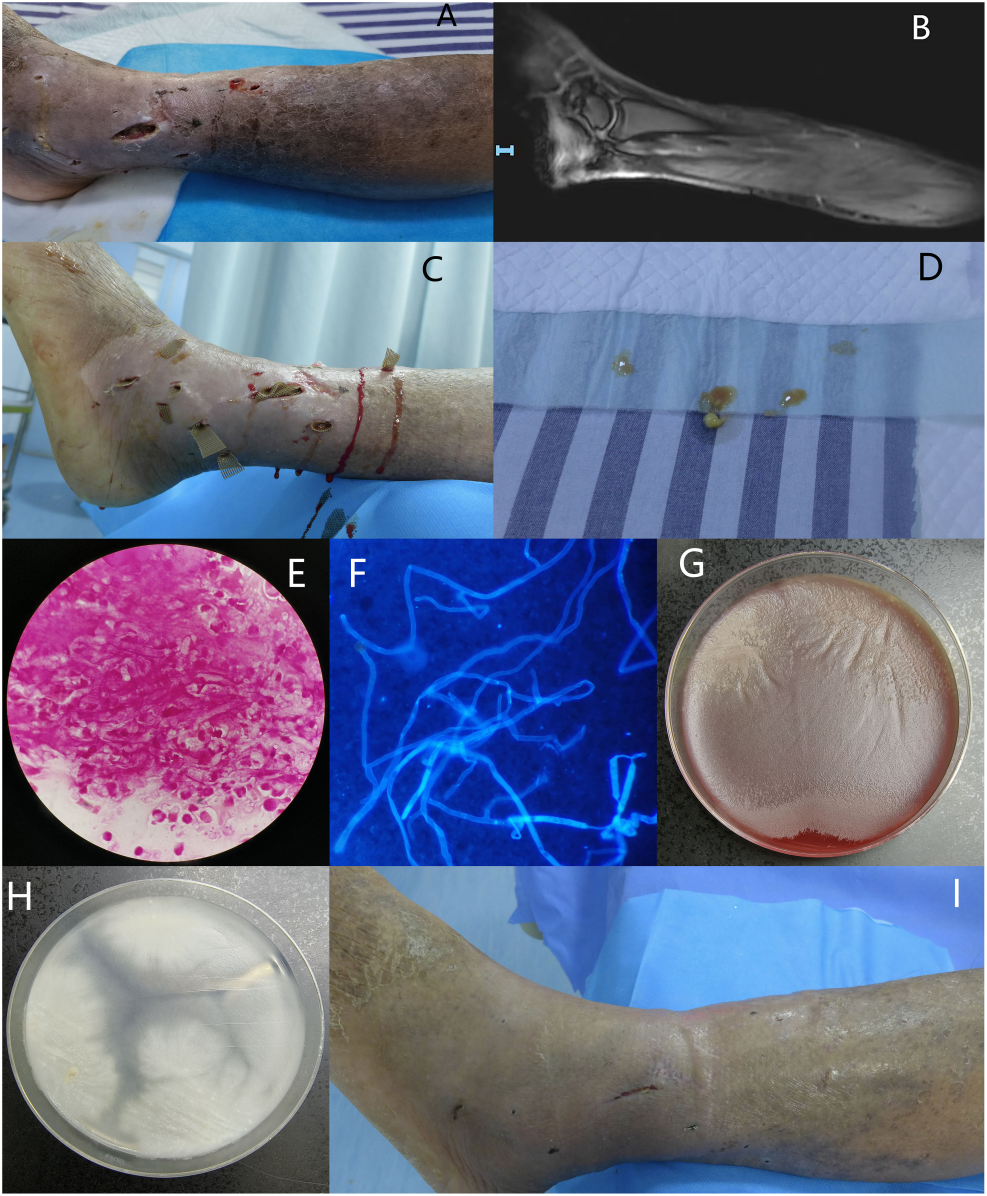
**(A)** Severe swelling, multiple ulcers and purulent secretions of the left lower limb, with scattered darkening indurations were observed and skin temperature of the indurated area was elevated; **(B)** Magnetic resonance imaging showed abnormal signals in various muscle groups and subcutaneous soft tissues of the left leg, indicating inflammatory lesions and abscesses. Increased signals were observed in the ligaments and tendons of the left foot, indicating inflammatory lesions and surrounding soft tissue swelling; **(C)** Subcutaneous abscesses debridement and drainage; **(D)** A significant amount of yellow purulent secretion was drained; **(E, F)** Gram staining and calcofluor white fluorescent staining revealed broad, irregularly branched, ribbon-like hyphae, ×1000; **(G)** Flat greyish-white colony on blood agar with filiform margins incubated at 35°C for 72 hours; **(H)** White to yellow-white, radiating colonies on PDA with few aerial hyphae incubated at 35°C for 72 hours; **(I)** After 24 days of treatment, most skin ulcers have successfully healed.

A 67-year-old man presented to our hospital due to leg skin ulcers for 20 days. He had contacted with swamp water after being bitten by ants. Also, the patient had diabetes, liver cirrhosis, and psoriasis. Physical examination showed severe swelling, multiple ulcers and purulent secretions of the left lower limb. Scattered darkening indurations were observed and skin temperature of the indurated area was elevated (**Figure 1A**). Laboratory findings included leukocyte 7.20×10^9/L, anemia (Hb: 73g/L), thrombocytopenia (85×10^9/L), hyperglycemia (7.05mmol/L), hypoproteinemia (TP: 48.9g/L; Alb: 22.9g/L) and EBV-DNA significantly increased (8.76×10^6 IU/mL). Elevated inflammatory markers included C-reactive protein 67.89mg/L, pocalcitonin 0.76 ng/mL, ESR 57.00mm/h. Magnetic resonance imaging showed abnormal signals in various muscle groups and subcutaneous soft tissues of the left leg, indicating inflammatory lesions and abscesses. Increased signals were observed in the ligaments and tendons of the left foot, indicating inflammatory lesions and surrounding soft tissue swelling (**Figure 1B**). Local debridement and abscess incision drainage were performed after admission (**Figure 1C**), and a significant amount of yellow purulent secretion was drained and sent for culture (**Figure 1D**).

On the second day of admission, Gram staining and calcofluor white-fluorescent staining of the wound secretion revealed broad, irregularly branched, ribbon-like hyphae, highly suggestive of fungal infection (**Figures 1E, F**). Itraconazole (200mg, once a day) for antifungal treatment and moxifloxacin (0.4g, once a day) for antibacterial treatment were initiated empirically. Flat greyish-white colony on blood agar with filiform margins and white to yellow-white, radiating colonies with few aerial hyphae on potato dextrose agar (PDA) were observed after 72 hours culture at 35°C (**Figures 1G, H**). Lactophenol cotton blue staining of the colonies showed broad, irregularly branched hyphae. Day 5 after admission, multiple isolated and unconnected subcutaneous abscesses appeared and subcutaneous pus were sent for metagenomic Next-Generation Sequencing, and the result was reported *P. insidiosum*. Thus, itraconazole was switched to linaconazole (600mg, once a day). After two weeks of treatment with linaconazole, the patient’s platelet count decreased to 26×10^9/L, which was considered as an adverse reaction to linaconazole, and it was discontinued. Minocycline (100mg, twice a day) was then used in combination with moxifloxacin. After 24 days of treatment, most skin ulcers have successfully healed (**Figure 1I**). The patient was discharged with prescriptions of continue oral minocycline 100mg, twice daily.

#### Patient 2

**Figure 2:**
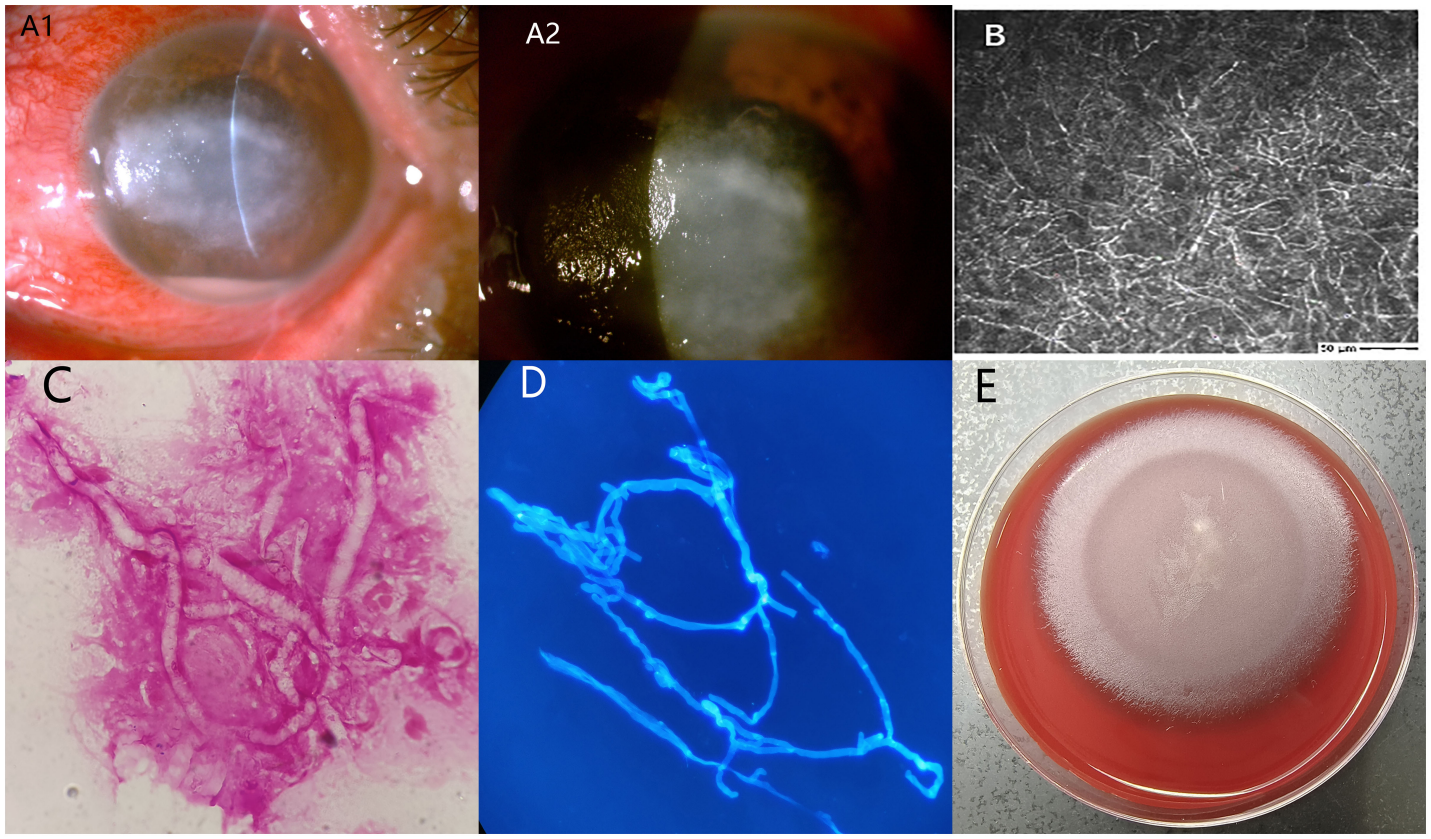
**(A1, A2)** The left eye showed dense central stromal opacity surrounded by a reticular pattern of subepithelial and superficial stromal infiltration; **(B)** Confocal microscopy displayed a large amount of filamentous structures at the lesion of the left cornea; **(C, D)** Corneal scraping with Gram staining and calcofluor white fluorescent staining revealed broad, irregularly branched, ribbon-like hyphae, ×1000; **(E)** Corneal scraping culture showed flat greyish-white colony on blood agar with filiform margins incubated at 35°C for 48 hours.

A 50-year-old man presented with pain in her left eye after being exposed to contaminated water in a rice field. The left eye showed dense central stromal opacity surrounded by a reticular pattern of subepithelial and superficial stromal infiltration (**Figures 2A1, A2**). Confocal microscopy displayed a large amount of filamentous structures at the lesion of the left cornea (**Figure 2B**). Corneal scraping with Gram staining and calcofluor white-fluorescent staining revealed broad, irregularly branched, ribbon-like hyphae (**Figures 2C, D**). Corneal scraping culture showed flat greyish-white colony on blood agar with filiform margins incubated at 35°C for 48 hours (**Figure 2E**). The organism was identified as *P. insidiosum* by zoospores production and metagenomic Next-Generation Sequencing. Unfortunately, due to the patient’s refusal of hospitalization and treatment, we were unable to track the prognosis of this patient.

### Strain identification

The isolates obtained were identified by examination of colonies morphology and microscopic feature. The first case’s subcutaneous pus and the second case’s isolate were sent for metagenomic Next-Generation Sequencing.

### Culture of *P. insidiosum* for zoospore detection

The colonies from the blood agar plates were used to induce zoospores for the purpose of microbiologically confirming *P. insidiosum*. A sterile petri dish was filled with sterile pond water, and a few sterile blades of echinochloa crus-galli (approximately 10mm long) were added. 2-3 pieces of agar blocks with *P. insidiosum* were placed in the petri dish, and the dish was incubated at 35°C for 48 hours. After changing fresh sterile pond water 1 hour, zoosporangiums begin to form on the mycelium. Once fully formed, the biflagellate zoospores break the zoosporangium wall, swim and then encyst about 30 min later. zoosporangiums and zoospores were observed on direct microscopic examination. The flagella of motile zoospores were looked for using Giemsa staining and scanning electron microscopy (SEM).

### Sample processing for mNGS

The subcutaneous pus was surgically removed from the skin ulcer and stored in sterile Phosphate Buffered Saline. Then subcutaneous pus was homogenized by vortex for 30 s and centrifuged at 15,000 rpm for 3 min and the supernatant was used for mNGS. The colony of corneal sample grew well on blood agar plate and the resulting colonies were collected and used for mNGS.

### Metagenomic sequencing

DNA extraction was performed following JIANSHI BIOTECH Universal DNA/RNA extraction protocol. Library prepared following KingCreate Biotechnology Pathogenic Microorganism Metagenomic DNA Detection Protocol (Miao et al., 2018). Both nucleic acid extraction and library preparation were conducted in parallel with quality control samples. Single-end 75bp sequencing was carried out using Illumina Nextseq 550 System with 75 cycles Reagent Kit. After filtering the low-quality sequencing data by fastp v0.20.0 (Chen et al., 2018) and removing the sequences mapped to the human reference genome using bwa v0.7.10-r789 (Li and Durbin, 2009), the remaining data were aligned to the National Center for Biotechnology Information (NCBI) GenBank database.

### Nucleotide identity analysis

The sequences obtained were analyzed using the National Center for Biotechnology Information GenBank database to identify microbial species.

### Physiological and morphology studies

Two strains were subcultured on blood agar (BA), Sabouraud medium (SDA) supplemented with chloramphenicol, potato dextrose agar (PDA), and CHROMagar Candida and incubated at 35°C, ambient air to observe growth on different media. Growth rates at different temperatures (25°C and 37°C) on BA, PDA incubated 24 hours, and SDA (without chloramphenicol) incubated for 4d.

### Antimicrobial susceptibility testing

By Clinical and Laboratory Standards Institute (CLSI) M38M51S-Ed3 and M100Ed32 protocol, the broth microdilution method and disk diffusion were used for susceptibility testing meanwhile. Zoospores inoculum was obtained by aforementioned zoosporogenesis technique. Upon encystment, the zoospores lose their flagella and become spherical. Zoospores do not produce turbidity as the conidia of filamentous fungi do. Thus, they were counted using a Neubauer chamber. Diluted in RPMI 1640 broth containing L-glutamine and buffered to pH 7.0, yielding a final concentration of 0.4×10^4^~5×10^4^ zoospores/mL for the broth microdilution method and 0.4×10^6^~5×10^6^ zoospores/mL for the disc diffusion method. All experiments were performed in duplicate.

For disk diffusion method, 200 μL of zoospores suspension was spread on the entire surface of a non-supplemented Mueller Hinton agar plate (for antifungal drugs) and Blood Mueller-Hinton with 5% sheep blood agar plate (for antibacterial drugs) by evenly streaking the swab over the entire agar surface. For the broth microdilution method, antimicrobial drugs concentrations ranged from 0.25μg/mL to 32μg/mL. All the plates and microdilution trays were incubated at 35°C, ambient air. The inhibition zone and the MIC were read after 48 hours. MICs were determined by visual observation of 100% growth inhibition compared to the growth in the control well containing no antimicrobial agent, after 48 h of incubation at 35°C. *Candida parapsilosis* (ATCC 22019) was used as controls for the antifungal drugs susceptibility testing. *Escherichia coli* (ATCC 25922) and *Staphylococcus aureus* (ATCC 25923) were used as controls for the for antibacterial drugs susceptibility testing.

23 antimicrobial agents were tested, including antifungal drugs of amphotericin B (AMB), flucytosine (FLU), itraconazole (ITR), voriconazole (VOR), posaconazole (POS), caspofungin (CAS), and antibacterial drugs of ceftriaxone (CRO), meropenem (MEM), cefepime (FEP), aztreonam (ATM), cefoperazone sulbactam (SCF), piperacillin tazobactam (TZP), vancomycin (VA), levofloxacin (LEV), ciprofloxacin (CIP), gentamicin (GEN), trimethoprim-sulfamethoxazole (SXT), clindamycin (DA), linezolid (LZD), erythromycin (E), azithromycin (AZM), minocycline (MH) and tigecycline (TGC). For disk diffusion method, antifungal drugs were obtained from Sigma Chemical Co. and antibacterial drugs from Oxoid, UK. For the broth microdilution method, all antimicrobial agents susceptibility kits were purchased from Zhuhai DL Biotech. Co., Ltd, China.

## Results

**Figure 3:**
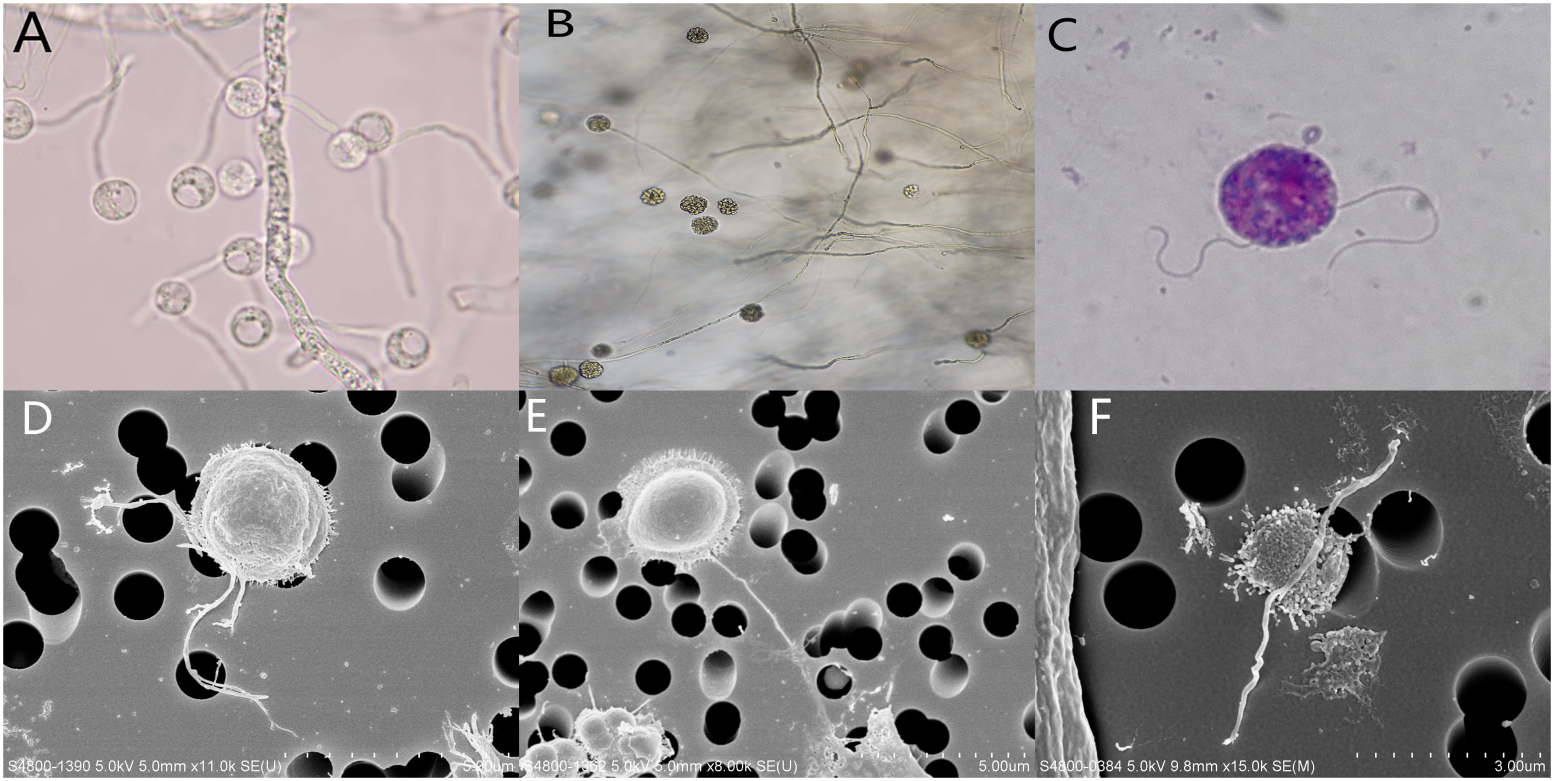
**(A)** sporangium was observed on direct microscopic examination, ×400; **(B)** Zoosporangium as observed on direct microscopic examination ×400; **(C)** Giemsa staining of zoospore showed two unequal flagella, ×1000; **(D–F)** Scanning electron microscopy of zoospore showed about 2.5μm in diameter, villose, kidney-like in shape, and two flagella arise from inside a lateral groove.

Lactophenol cotton blue staining of the colonies showed broad, irregularly branched hyphae. In water culture, sporangium (**Figure 3A**) and large zoosporangium (20 to 60 µm in diameter, **Figure 3B**) were observed after induction, zoospores are released after rupture of the zoosporangium membrane. Giemsa staining of zoospores showed two unequal flagella (**Figure 3C**). Scanning electron microscopy of zoospore showed about 2.5μm in diameter, villose, kidney-like in shape, and two flagella arise from inside a lateral groove (**Figures 3D–F**). The obtained mNGS nucleotide sequences of two cases were compared with the nearest sequence at the National Center for Biotechnology Information GenBank database. The first homology sequence presenting the highest identity of patient 1 and patient 2 were 100% nucleotide identity with *Pythium insidiosum* isolate GZ2020 (GenBank No. JAKVDI010000034.1).

Colonies on blood agar are white to pale yellow, flat or slightly concave, radial, and typically lack aerial hyphae. The mycelium has thin walls, irregular thickness, is readily folded, and is sparsely septate. The growth rate on blood agar was faster than PDA plates, and no colony growth was observed on SDA and CHROMagar Candida (supplemented with chloramphenicol) incubated at 35°C for 24 hours. The results showed that the strain could grow at 25°C and 37°C temperatures, with the 37°C growth rates faster than 25°C on BA. The growth rate of colonies on SDA (not supplemented with chloramphenicol) and PDA does not differ significantly at 25°C and 37°C.

**Figure 4:**
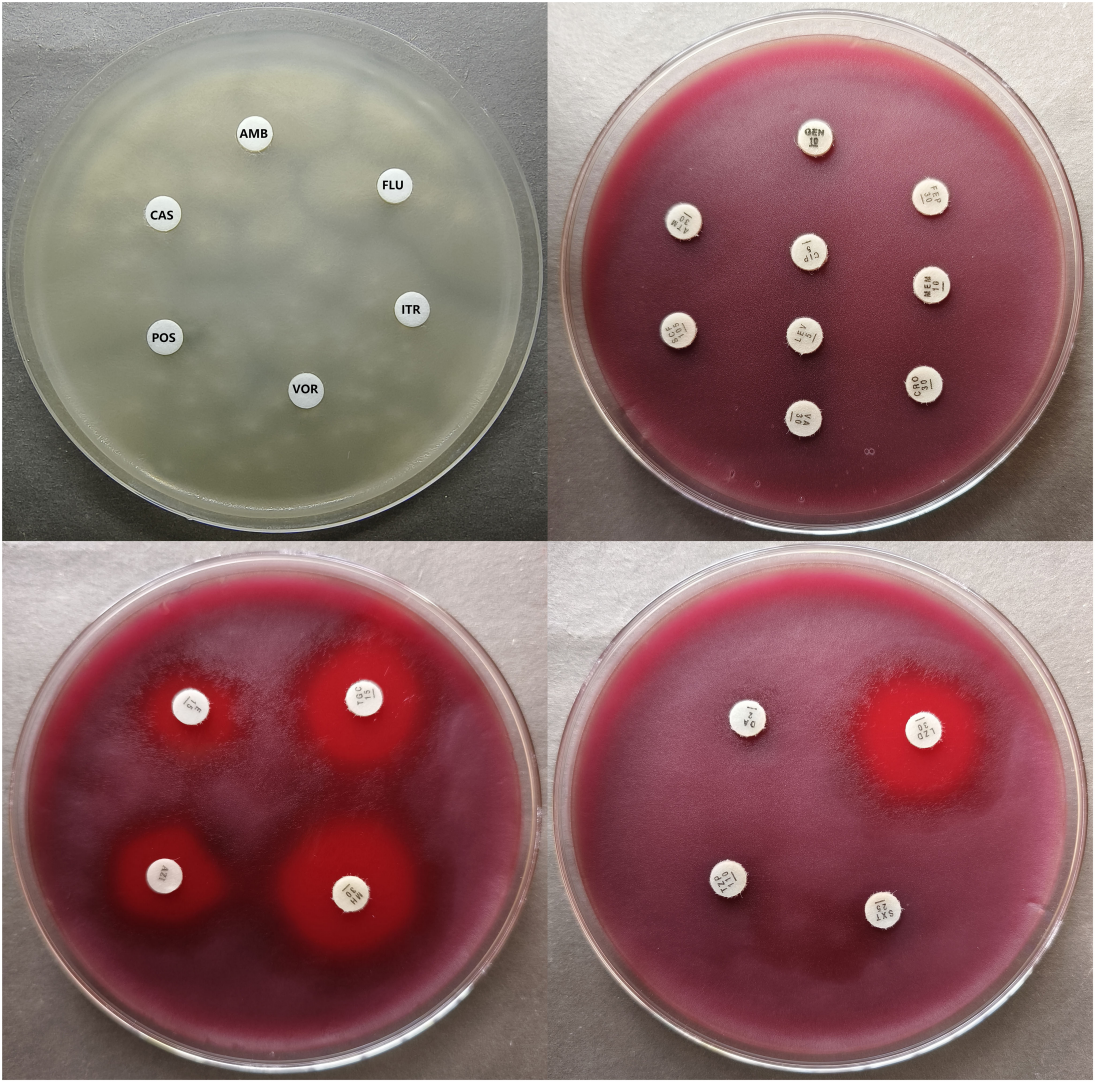
Inhibition zone of the strain 1 after incubating at 35°C for 48 hours.

All antifungal drugs showed no inhibition zones for *P. insidiosum* and the MICs were >32μg/mL, which indicated antifungal drugs have no inhibitory activity against *P. insidiosum in vitro*. (**Table 1**). Antibacterial drugs of β-Lactams (ceftriaxone, meropenem, cefepime, aztreonam, cefoperazone-sulbactam, Piperacillin-tazobactam), vancomycin, levofloxacin, ciprofloxacin, gentamicin, trimethoprim-sulfamethoxazole, clindamycin also showed no inhibition zones and the MICs were >32μg/mL (**Table 2**), which proved that the above-mentioned drugs have no inhibitory activity against *P. insidiosum in vitro*. MICs of strain 1 and strain 2 for erythromycin were 4μg/mL, for azithromycin was 1μg/mL and 2μg/mL, respectively, for minocycline were 1μg/mL, for tigecycline was 1μg/mL and 0.5μg/mL, respectively, and for linezolid was 0.5μg/mL and 1μg/mL, respectively (**Table 2**). Minocycline, tigecycline, and linezolid showed significantly inhibition zones (**Table 2**). The inhibition zones of erythromycin and azithromycin were smaller than minocycline, tigecycline, and linezolid (**Table 2**). Inhibition zones of the strain 1 after incubating at 35°C for 48 hours were showed in **Figure 4**. The results showed that minocycline, tigecycline, linezolid, erythromycin and azithromycin have significant *in vitro* activity against *P. insidiosum*.

**Table 1:** *In vitro* susceptibility testing of the antifungal drugs against *P. insidiosum*.

Drug name	strain 1		strain 2	
	MIC (μg/mL)	Inhibition zone (mm)	MIC (μg/mL)	Inhibition zone (mm)
Fluconazole	>32	6	>32	6
Itraconazole	>32	6	>32	6
Voriconazole	>32	6	>32	6
Posaconazole	>32	6	>32	6
Amphotericin B	>32	6	>32	6
Caspofungin	>32	6	>32	6

**Table 2:** *In vitro* susceptibility testing of the antibacterial drugs against *P. insidiosum*.

Drug name	strain 1		strain 2	
	MIC (μg/mL)	Inhibition zone (mm)	MIC (μg/mL)	Inhibition zone (mm)
Minocycline	1	25	1	24
Tigecycline	1	21	0.5	23
Azithromycin	1	16	2	15
Erythromycin	4	14	4	14
Linezolid	0.5	26	1	24
Aztreonam	>32	6	>32	6
cefoperazone sulbactam	>32	6	>32	6
Gentamicin	>32	6	>32	6
ciprofloxacin	>32	6	>32	6
Levofloxacin	>32	6	>32	6
Vancomycin	>32	6	>32	6
Cefepime	>32	6	>32	6
Meropenem	>32	6	>32	6
Ceftriaxone	>32	6	>32	6
Piperacillin tazobactam	>32	6	>32	6
Clindamycin	>32	6	>32	6
Trimethoprim-sulfamethoxazole	>32	6	>32	6

## Discussion

Treatment success for pythiosis depends on an early and precise diagnosis. However, medical practitioners’ inexperience of pythiosis hinders prompt and accurate diagnosis, which may lead to incorrect or delayed strategies for treatment. Currently, direct microscopic examination, microbial culture, and induced zoospores assays are the main detection methods. *P. insidiosum* presented wide, sparsely septate mycelial structure under direct microscopic examination, which is easily misdiagnosed as fungi. The organism is easily mistaken for contaminating fungi and discarded, or is treated incorrectly as fungi resulting in delayed therapy. Zoosporangium and zoospores are characteristic structures of *P. insidiosum*, which distinguish it from fungi. *P. insidiosum* grows well on blood agar and potato dextrose agar, but does not grow or grows poorly on SDA plate supplemented with chloramphenicol. The colonies are white or yellow-white, with concave growth, wavy or radiating appearance, and rarely have aerial mycelium. Failure to isolate *P. insidiosum* may be due to the low-temperature storage of specimens, as the optimal growth temperature for *P. insidiosum* is 28°C to 32°C, and isolate growth was completely inhibited at 8°C (Krajaejun et al., 2010). At 42°C, most isolates failed to grow, whereas a few isolates grew minimally (Krajaejun et al., 2010). The serological diagnosis of pythiosis usually relies on immunodiffusion tests. Although immunodiffusion tests have high specificity, their sensitivity is poor (Pracharktam et al., 1991). Subsequently, other diagnostic methods have been developed, such as enzyme-linked immunosorbent assay (ELISA) (Krajaejun et al., 2002), immuno-chromatographic tests (Krajaejun et al., 2009), western blotting (Krajaejun et al., 2006a), and PCR assays (Gaastra et al., 2010), which have good specificity and sensitivity. Nevertheless, because pythiosis is rare in China, serological diagnostic testing is not performed commonly because most Chinese hospitals lack the necessary diagnostic supplies and technology. Additionally, such immunological tests cannot diagnose patients with localized infections (e.g., ocular infections). Nucleic acid-based detection (NAT) can effectively and directly detect low levels of *P. insidiosum* DNA in either trace amounts or culture-negative samples (Sridapan and Krajaejun, 2022). Metagenomic Next-Generation Sequencing is a novel, fast, and robust microbiological detection method, especially in emergency clinical situations where the pathogens are unknown (Sridapan and Krajaejun, 2022). However, this technique is costly and many laboratories are unable to use it, currently limiting its use as a routine microbiological detection analysis (Sridapan and Krajaejun, 2022).

Human pythiosis cases have increased dramatically during the past ten years, infection occurs when an individual has a skin wound or abrasion that comes into contact with the zoospores within the contaminated water (Gaastra et al., 2010). Agricultural related activities or water associated leisure activities are considered to be predisposing factors for human pythiosis (Gaastra et al., 2010). So far reported, there are three *Pythium* species affecting mammalian hosts: *P. aphanidermatum*, *P. insidiosum* and *Pythium periculosum* (Miraglia et al., 2022). Among them, *P. insidiosum* is the most common species. The diverse range of *Pythium* species can greatly affect the epidemiology, diagnosis and management of their infections, especially in the performance of serological diagnosis and effective implementation of immunotherapy of pythiosis (Miraglia et al., 2022). Cutaneous/subcutaneous abscesses caused by *P. insidiosum* may present with various skin manifestations, such as vesicles/bullae, skin ulcers, cellulitis, chronic swelling, subcutaneous lesions, infiltrative masses and ulcers (Gurnani et al., 2022b). If left untreated, cutaneous/subcutaneous infections may progress to vascular abscesses, leading to limb amputation (Gurnani et al., 2022b). Several studies in Thailand showed that almost all patients with Cutaneous/subcutaneous, vascular, and disseminated pythiosis have underlying hemoglobinopathy-Mediterranean anemia syndrome or blood system diseases including paroxysmal nocturnal hemoglobinuria, aplastic anemia, myeloproliferative disorders, idiopathic thrombocytopenic purpura, and leukemia (Wanachiwanawin et al., 1993; Gurnani et al., 2022b; Chitasombat et al., 2020). Most ocular cases were associated with no underlying diseases (Gurnani et al., 2022b). In our first case, the patient’s left lower limb was infected during contact with swamp water after being bitten by ants. The patient presented with rapidly worsening cutaneous abscesses and ulcers. Decompensated cirrhosis, diabetes, and psoriasis may be potential predisposing factors for pythiosis in the patient.

Most cases of *Pythium* keratitis have a history of trauma that occurred before (Gurnani et al., 2021). It is difficult to distinguish from fungal keratitis during a slit-lamp examination without a high degree of clinical suspicion. The presence of patchy reticular dot-like subepithelial and stromal infiltrates, multiple infiltrates dispersed throughout the cornea and a stromal infiltrate with hyphated edges that resembles cotton wool are among the distinctive clinical features that differentiate it from fungal keratitis (Anitha and Vanathi, 2021; Gurnani et al., 2022a). Recent *in vitro* research has led to the recommendation that antibiotics, such as 1% azithromycin and 0.2% linezolid, be used as first-line medications for *Pythium* keratitis (Gurnani et al., 2022a). However, many cases of *Pythium* keratitis may not respond well to pharmaceutical treatment because of its high virulence, recurrence rate, and capacity for rapid proliferation. In nonresolving cases, early therapeutic keratoplasty is necessary (Gurnani et al., 2022a). In any case, a proper diagnosis without delay and prompt treatment with the right medications are essential for successful patient management of *Pythium* keratitis. For our *Pythium* keratitis case, the patient discontinued the follow-up at the eye clinic. So, its clinical outcome could not be followed although early and correct diagnosis was given.

The treatment for abscesses caused by *P. insidiosum* involves surgical intervention, medication, and immunotherapy. The key to controlling the disease is a combination of immediate surgical intervention and aggressive medication treatment. Anti-fungal drugs are usually ineffective against *P. insidiosum* because it does not synthesize ergosterol (Lerksuthirat et al., 2017). A Study showed that itraconazole and terbinafine have synergistic inhibitory effects on the *in vitro* growth of *P. insidiosum* and long-term combination therapy with itraconazole and terbinafine has successfully cured *P. insidiosum* infection (Shenep et al., 1998). Heath, JA, et al. have successfully cured leukemia patients with *P. insidiosum* pleuropericarditis using liposomal amphotericin B and itraconazole (Heath et al., 2002). However, several studies have shown that antifungal drugs are ineffective against *P. insidiosum* (Sathapatayavongs et al., 1989; Gurnani et al., 2022b), and Bhupesh Bagga, et al. have compared the rate of therapeutic penetrating keratoplasty (TPK) and proportion of healed ulcers in the group on antifungal therapy (TPK—11/13, 84.6%; Healed—2/13, 15.3%) with the group on antibacterial therapy (TPK—11/17, 64.7%; Healed—6/17, 35.2%). The former has higher rate of TPK and lower proportion of healed ulcers (p=0.21, Fisher’s exact test) (Bagga et al., 2018). Our case suggested that antifungal drugs have no activity *in vitro* against *P. insidiosum*.

In a study on *in vitro* drug sensitivity of pythium, antibiotics such as miltefosine, azithromycin, clarithromycin, josamycin, linezolid, and sutezolid showed good activity and are promising candidate drugs for humans pythiosis (Loreto et al., 2018). Macrolides and tetracycline antibiotics also show good activity to *P. insidiosum*, with minocycline being considered the most effective drug in testing (Loreto et al., 2011). In our case, we have determined the activity of 17 antibacterial and 6 antifungal drugs against *P. insidiosum* using broth microdilution and disk diffusion methods *in vitro*. The lowest MICs and the largest zones of inhibition (disk diffusion) were observed for minocycline, tigecycline and linezolid. All of the zones of inhibition and the MICs were close to the previous studies (Yolanda and Krajaejun, 2020). Up to now, no standard CLSI guideline protocol for *in vitro* drug susceptibility of *P. insidiosum* is available. For better interpretation and application of susceptibility test data, it is very necessary to standardize the susceptibility method and correlating drug susceptibility data with clinical outcome of human pythiosis. Maeno et al. successfully treated a 20-year-old Japanese male with *P. insidiosum* keratitis using a combination of minocycline, linezolid, and chloramphenicol (Maeno et al., 2019). Two cases of keratitis were also successfully cured using a combination of linezolid and azithromycin (Gurnani et al., 2022b, 2023).

In our first case of cutaneous abscess, liver cirrhosis decompensation, diabetes, and psoriasis were considered as potential triggering factors for *P. insidiosum* infection. Clinicians should have a high suspicion of cutaneous/subcutaneous pythiosis in immunocompromised patients with a history of sewage exposure. It is crucial for patient prognosis. Once diagnosed, patients should receive appropriate treatment, including the use of linezolid, macrolides, minocycline, and tigecycline, which have been proven effective in current research, along with aggressive surgical debridement. Our cases provide a reference for future studies to determine the optimal treatment for cutaneous and subcutaneous pythiosis in immunocompromised patients.

## Conclusions

Early and accurate identification, combined with aggressive surgical debridement and appropriate drug therapy, can greatly improve patient managements. Conventional culture and zoospore induction remain gold standard for diagnosis; however, DNA-based method should be performed simultaneously. The drug susceptibility testing provides profound effects on proper drug selection against *P. insidiosum*.

## Data Availability

The data has been deposited into the National Center for Biotechnology Information GenBank database. GenBank accession number were PQ182808-PQ182826.
